# The Relationship of Pork Meat Consumption with Nutrient Intakes, Diet Quality, and Biomarkers of Health Status in Korean Older Adults

**DOI:** 10.3390/nu16234188

**Published:** 2024-12-04

**Authors:** Ah-Jin Jung, Anshul Sharma, Mei Chung, Taylor C. Wallace, Hae-Jeung Lee

**Affiliations:** 1 Department of Food and Nutrition, Gachon University, Seongnam-si 13120, Republic of Korea; dkwls2784@naver.com (A.-J.J.); anshul.silb18@gmail.com (A.S.); 2 Institute for Aging and Clinical Nutrition Research, Gachon University, Seongnam-si 13120, Republic of Korea; 3 Gerald J. and Dorothy R. Friedman School of Nutrition Science and Policy, Tufts University, Boston, MA 02111, USA; mei_chun.chung@tufts.edu (M.C.); taylor.wallace@me.com (T.C.W.); 4 Think Health Group, LLC., Washington, DC 20001, USA; 5 School of Medicine and Health Sciences, George Washington University, Washington, DC 20052, USA; 6 Department of Health Sciences and Technology, GAIHST, Gachon University, Incheon 21999, Republic of Korea; 7 Clinical Research Center, Gachon University Gil Medical Center, Incheon 21565, Republic of Korea

**Keywords:** pork, meat, diet, biomarkers, nutritional status, Republic of Korea

## Abstract

Background: Pork meat is a widely consumed protein food with the potential to differentially affect health and nutritional status across social and cultural contexts. Objectives: We evaluated the association between pork meat consumption and nutrient intake, diet quality, and biomarkers of health among older adults (age ≥ 65 years) in Korea. Methods: Our analyses utilized dietary and health examination data from the 2016–2020 Korean National Health and Nutrition Examination Survey (*n* = 2068). Comparisons between variables derived from the nutrition survey and health examination by pork consumption (consumers vs. non-consumers) were assessed using regression analyses for survey data. Results: Pork consumption was found to be associated with younger age, greater educational attainment, and lower likelihood of living in a rural area. Consumption was also associated with a higher intake of energy and all nutrients except vitamin B6, retinol, ⍵3, and zinc in males and vitamin B6 in females. Diet quality was modestly higher among male (67.91 ± 0.93 vs. 65.74 ± 0.74; *p* = 0.0308) and female (70.88 ± 0.96 vs. 67.00 ± 0.73; *p* < 0.0001) pork consumers. Differences in biomarkers were clinically irrelevant, with inconsistencies between genders. Handgrip strength was slightly higher among male (33.84 ± 0.52 vs. 31.91 ± 0.40; *p* < 0.0001) and female (20.76 ± 0.34 vs. 19.99 ± 0.22; *p* < 0.0001) pork consumers. Conclusions: In Korean older adults, pork consumption may contribute to a higher intake of energy and most nutrients, improved diet quality scores, higher vegetable intake, and small improvements in health biomarkers. Further well-designed studies are needed to confirm these findings.

## 1. Introduction

Pork is a flavorful and widely consumed protein food that is a source of many essential nutrients and is integral to the diet of most cultures worldwide [1]. Global pork demand was predicted to reach 128.9 million metric tons on an annual basis in 2022 [2]. Observational data that isolate how pork may impact the diet and health of various populations are limited, as pork is often grouped with other red meats [3]. However, pork is distinct from other red meats because of its increased acceptance across various cultures, distinct flavor profile, lower carbon footprint, and greater protein digestibility [3,4,5].

Consumption patterns and foods co-consumed with pork have also been suggested to differ substantially across cultures. In the United States, adults who consume pork largely report an intake of processed pork (87%), whereas only a small proportion report an intake of fresh–lean pork (9%) [6]. Condiments and white bread are popular foods co-consumed with pork in the United States and, therefore, negatively impact its relationship with overconsumed nutrients and food groups such as sodium, added sugars, and refined grains [6]. The Asian region has experienced the third largest increase in red and processed meat intake over the last decade, suggesting a transition toward a more Western dietary pattern [7]. However, various aspects of traditional dietary patterns in Asia have been preserved, such as a high vegetable and plant-food intake in South Korea [8,9,10]. Asian cultures also tend to consume fresh cuts and very little processed pork. In Japan, there is a preference for leaner cuts, as the popular dishes Tonkatsu and Shabu Shabu are prepared with fresh cuts of Boston butt and loin [10].

South Korea continues to be one of the world’s top pork-consuming nations, with annual per capita consumption rates rising from 22.8 to 28.5 kg per person between 2015 and 2022, respectively [11]. South Koreans have a greater preference for fresh cuts of pork belly [10]. However, pork composition differs significantly based on cuts, origins, animal nutrition, and preparation methods, all of which vary in terms of nutritional value and potential health effects [12]. According to 2024 data from the Korean Food Composition Database, the nutritional composition of different pork cuts, including belly, ham, loin, and tenderloin, varies in terms of their cooked (roasted and pan-broiled) and raw forms [13]. For instance, pork belly in its raw and roasted forms has the highest fat content (33.3 and 41.2 g/100 g) and the lowest protein content (13.9 and 22.78 g/100 g), respectively. Meanwhile, the fat content of ham, loin, and tenderloin varies from 3.15 to 4.97 g/100 g in their raw forms to 4.76 to 8.93 g/100 g in the pan-broiled state. The protein content in raw and pan-broiled versions of ham, loin, and tenderloin varies from 20.88 to 23.33 g/100 g to 30.21 to 32.06 g/100 g, respectively [13]. The presence of unfavorable saturated fatty acids (SFAs) and heme iron content in pork belly cuts has been suggested to increase consumer risk of cancer [14] and dyslipidemia [15]. In this regard, the Ministry of Agriculture, Food, and Rural Affairs and the Ministry of Food and Drug Safety of Korea collaborate in order to develop new frameworks that enable the processing of food products using low-fat cuts. They also engage in advertising efforts to raise awareness among consumers about a healthy diet that utilizes low-fat pork cuts [16].

The older adult population is increasing faster in South Korea than in any other country, indicating a demographic change from an aging population to an aged population [17]. According to national statistics from the South Korean government, the population aged ≥65 years increased from 7.07 million in 2017 (13.8% of the population) to 8.15 million (15.7% of the population) in 2020 [18]. South Korea is predicted to be considered a super-aged society, defined as having ≥20.0% of its total population aged ≥65 years, by 2025 [17]. An important contributing factor is the increasing life expectancy of South Koreans, which is 80.3 and 86.3 years for males and females, respectively, according to 2019 Korean statistics [19]. In a 2020 survey, 72.2% of older adults in Korea had adequate nutritional management; nonetheless, 19% required attention and 8.8% needed improvement [20].

According to a study based on data from the Sixth Korean National Health and Nutrition Examination Survey (KNHANES) (2013–2014), more than half of Koreans aged >60 years consume less protein than the recommended dietary allowance (RDA) [21]. In an examination of the protein intake trends of Koreans from 2010 to 2019, the rate of protein intake below the estimated average requirement (EAR) was highest in both men and women aged >65 years compared with other age groups. The proportion of people eating less than the EAR was 34.5% for older men and 44.7% for older women in 2019 [21]. According to the 2020 Dietary Reference Intake for Koreans, the recommended daily protein intake is 60 g and 50 g for older males and females (>65 years), respectively [22]. Plant-based proteins account for the majority of protein intake in Korea, with animal proteins accounting for about one-third of the total RDA [23]. Korean national dietary guidelines emphasize the importance of consuming protein-rich food such as meat, fish, eggs, and beans every day; however, the risk of inadequate intake remain high among older adults [24]. Consumption of animal protein has been reported to be associated with muscle mass and is helpful for sarcopenia [25,26]. A 2020 analysis of the U.S. population found pork intake to be associated with increases in total energy and intake of most nutrients but decreases in diet quality. Fresh and fresh–lean pork has been suggested to lessen the likelihood of functional impairment among U.S. adults aged ≥65 years [27]. Despite the increased pork consumption (particularly of fresh cuts) in South Korea, to our knowledge, there is a dearth of studies that assess potential associations of pork intake with diet and health in the Korean population, especially older adults. Thus, this study aimed to evaluate the relationship of pork consumption with total energy and nutrient intake, diet quality, and markers of health status among Korean adults aged ≥65 years (hereinafter, older adults) using data from the seventh and eighth KNHANES.

## 2. Methods

### 2.1. Study Design and Population

Data from the 2016–2020 KNHANES data cycles were utilized in these cross-sectional analyses. The KNHANES is an annual survey that was initiated in 1998 to assess the health and health-related behaviors of the Korean population. KNHANES participants are all family members aged ≥1 year in the selected primary sampling units and households. The survey is administered by the Korean Disease Control and Prevention Agency (KDCPA), which utilizes a stratified multistage probability sampling design and includes a health interview, health examination, and nutrition survey. A detailed description of the sample design, subjects, survey components, and survey methods can be found in the guidebook for the KNHANES database and in the scientific literature [28,29,30,31]. The Institutional Review Board of the KDCPA approved the KNHANES study protocol (2018-01-03-P-A, 2018-01-03-C-A, and 2018-01-03-2C-A).

**Figure 1:**
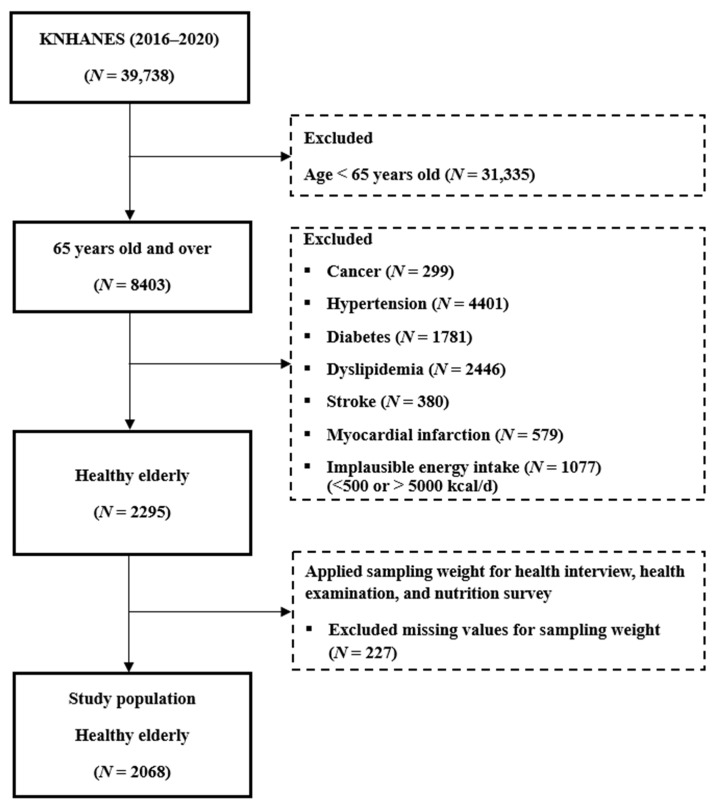
Flow diagram of inclusion and exclusion of study participants. KNHANES = Korean National Health and Nutrition Examination Survey.

A total of 39,738 individuals were enrolled in the 2016–2020 KNHANES data cycles, of which 8403 were aged ≥65 years. Individuals with a major chronic disease (e.g., cancer, hypertension, diabetes, dyslipidemia, stroke, or myocardial infarction), those with extreme energy intakes (<500 or >5000 kcal), and those missing data from the nutrition survey, health interview, or health examination were excluded from these analyses. There were a total of 227 missing values for sampling weight. Our final analytical sample included data from 2068 participants (Figure 1).

### 2.2. Development of a Vitamin B_6_, Vitamin B_12_, Magnesium, Zinc, and Selenium Database

We pooled data from various domestic and international food composition databases to generate a database that can be used to estimate the intakes of vitamin B_6_, vitamin B_12_, magnesium, zinc, and selenium from food intake reported in the KNHANES (2016–2020) 24 h dietary recall data. The steps for determining the nutrient value/edible 100 g were as follows:

1. Foods with the same description (exact same names [common or academic names] and same condition);
2. Foods with the same name but different water content (dried, raw, and boiled) or different manufacturer;
3. Substituting similar foods while considering major nutrients (specifically, carbohydrate, protein, and fat content).

Our database gave priority to domestic databases, with the Rural Development Administration Korean Food Composition database (2022) being the highest priority [32], followed by the Korean Ministry of Food and Drug Safety processed food database (2020) [33] then the Korean Nutrition Society CAN-Pro database [34]. In the absence of a suitable food match, we used international data. Databases from neighboring countries were used in the following order: the Japan Standard Tables of Food Composition [35] and the U.S. Department of Agriculture FoodData Central database [36]. Table S1 presents the number of foods in each database used to calculate vitamin B_6_, vitamin B_12_, magnesium, zinc, and selenium intake.

### 2.3. Estimation of Total Energy and Nutrient Intake

Total energy and nutrient intakes were derived from food intake reported in the 1-day (24 h) dietary recall. Macro- and micronutrients included carbohydrate, protein, fat, SFAs, omega-3 (⍵3) fatty acids, omega-6 (⍵6) fatty acids, cholesterol, sugar, minerals (calcium, phosphorus, magnesium, iron, zinc, sodium, potassium, and selenium), and vitamins (vitamins A, C, B_6_, and B_12_ and carotene, retinol, thiamin, riboflavin, niacin, and folic acid). Total energy and nutrient intakes, except for vitamin B_6_, vitamin B_12_, magnesium, zinc, and selenium, were obtained from the KNHANES data. Intakes of vitamin B_6_, vitamin B_12_, magnesium, zinc, and selenium were estimated using our newly developed database (described in Section 2.2).

### 2.4. Classification of Pork Consumers and Non-Consumers

We categorized pork intake from food items reported in KNHANES as fresh pork, processed pork, and total pork. Fresh pork refers to unprocessed raw pork. Processed pork includes bacon, ham, and sausage. Total pork includes both fresh and processed pork. Participants were categorized into two groups (pork consumers and non-consumers) based on their reported intake from the 24 h dietary recall. Both groups were further stratified by gender.

### 2.5. Korean Healthy Eating Index Score

The Korean Healthy Eating Index (KHEI) is an indicator score developed to evaluate overall diet quality and compliance with Korean national dietary guidelines [37]. The total KHEI score ranges from 0–100 and is derived through the summation of the following 14 component scores: (1) meat, fish, egg, and bean intake; (2) have breakfast; (3) mixed grains intake; (4) total fruit intake; (5) fresh fruit intake; (6) total vegetable intake; (7) vegetable intake, excluding kimchi and picked vegetables; (8) milk and milk products intake; (9) energy from SFA; (10) sodium intake; (11) energy from sweets and beverages; (12) percent energy from carbohydrate; (13) percent energy from fat; and (14) energy intake. A higher KHEI score indicates higher diet quality, while higher subcomponent scores indicate greater adherence to the individual components [37,38]. KHEI data are available for the 2016–2018 data cycles of the KNHANES (*n* = 1234). 

### 2.6. Anthropometric, Blood Pressure, and Biochemical Measurements

Data from the health examination of the KNHANES (e.g., biomarkers, anthropometric measurements, and blood pressure) were used to assess differences between pork consumers and non-consumers. Eight-hour fasting blood samples were collected from participants in the morning and were analyzed at the Seegene Medical Foundation (Seoul, Republic of Korea) within 24 h. Hemoglobin, hematocrit, blood glucose, high-sensitivity C-reactive protein (hs-CRP) (2016–2018 data cycles only), creatinine, blood urea nitrogen (BUN), total cholesterol (TC), high-density lipoprotein (HDL) cholesterol, low-density lipoprotein (LDL) cholesterol, and triglycerides (TG) were assessed using a Hitachi 7600–210 automatic analyzer (Hitachi, Tokyo, Japan) and the XN-9000 hematology system (Sysmex, Kobe, Japan). LDL cholesterol concentrations were calculated using Friedewald’s formula: TC – HDL cholesterol − TG/5. Glycated hemoglobin A_1c_ (HbA_1c_) levels were determined using G8 automated high-performance liquid chromatography (Tosoh, Tokyo, Japan). Anthropometric measurements, including height, weight, and waist circumference, and blood pressure (systolic blood pressure [SBP] and diastolic blood pressure [DBP]) were obtained using standardized methods.

### 2.7. Regular Physical Activity

We categorized participants who engaged in regular physical activity and those who did not. Regular physical activity was defined as “moderate-intensity activity for more than 150 min, a high-intensity activity for more than 75 min, or a combination of both moderate- and high-intensity activity (1 min for high intensity and 2 min for moderate intensity) per week”.

### 2.8. Handgrip Strength Measurements

Handgrip strength is an indicator of overall muscle strength and has been correlated with high protein intake in certain studies [39]. Participant handgrip strength was measured three times by crossing both hands from the dominant hand using a digital grip strength dynamometer (TKK-5401; Takei Scientific Instruments, Tokyo, Japan) [40,41]. Participants were asked to exert maximum force in a standing position with the forearm fully extended in a position away from the thigh level of the body. The maximum value among the measured values of both hands was used in this study. Hand grip strength was not measured in 2020 due to the COVID-19 pandemic. Low muscle strength was defined as handgrip strength of <28 kg for men and <18 kg for women [42].

### 2.9. Statistical Analyses

Statistical analyses were performed with SAS, version 9.4 (SAS Institute, Cary, NC, USA), and significance was set at *p <* 0.05. All analyses were conducted using the “PROC SURVEY” procedure by applying weights, given the complex sample design (cluster and strata). The Rao–Scott chi-square test was applied to assess differences in general characteristics between pork consumers and non-consumers within each gender. Comparisons between continuous variables derived from the nutrition survey and health examination by pork consumption (consumers vs. non-consumers) were assessed using the “PROC SURVEYREG” procedure, adjusting for age and gender. The mean values of age- and gender-adjusted continuous variables were assessed using analysis of covariance. Given the known differences in dietary patterns and nutrient intake between genders, data are reported separately for men and women. Results are reported as means ± SEs unless otherwise noted.

## 3. Results

### 3.1. Characteristics of Study Participants

The study population (*n* = 2068) had a mean age of 72.28 ± 0.13 years. Pork consumers (males and females) were younger in age, more likely to obtain a higher level of education, and more likely to live in an urban residence. Consumption was associated with higher household income and being an alcohol consumer in males only (no difference in females). Consumption was not associated with differences in marital status, smoking status, or physical activity in males or females (Table 1).

### 3.2. Proportion of Pork Intake

Total pork intake among participants in the KNHANES dataset was approximately 63.11 ± 3.16 g/d. Participants consumed predominantly fresh pork and only small amounts of processed pork. The intake amount was higher among males than females and highest among obese males (Table 2).

### 3.3. Total Energy and Nutrient Intake

Pork consumption was found to be associated with a higher intake of total energy, carbohydrate, protein, fat, SFA, ⍵6 fatty acids, cholesterol, sugar, calcium, phosphorus, iron, sodium, potassium, vitamin A, carotene, thiamin, riboflavin, niacin, folate, vitamin C, magnesium, selenium, and fiber in males and females. Pork consumption was only significantly associated with an increased intake of ⍵3 fatty acids, retinol, and zinc in females, with no significant association in males. Consumption was associated with a decrease in vitamin B_12_ intake in males and an increase in females. There was no association between pork intake and vitamin B_6_ intake in males or females (Table 3 and Table S2) 

### 3.4. Diet Quality

Pork consumption was found to be associated with a higher KHEI score, a higher item score for the “meat, fish, egg, and bean intake, “ “total fruit intake”, “total vegetable intake”, “percent energy from SFA”, and “percent energy from fat” items; a decreased item score for the “sodium intake” item; and no difference in item score for the “mixed grains intake”, “percent energy from sweets and beverages”, and “energy intake” items in both males and females. Consumption was associated with a lower item score for the “having breakfast” item, and no difference in the item score for the “fresh fruit intake”, and “milk and milk products intake” items in males. Consumption was associated with a higher item score for “having breakfast” and “fresh fruit intake” items, and lower item score for the “milk and milk products intake” and “percent energy from SFA” items in females (Table 4 and Table S3). 

### 3.5. Biomarkers of Health Status

Pork consumption was found to be associated with a small increase in hemoglobin, hematocrit, creatinine, total cholesterol, low-density lipoprotein cholesterol (LDL-c) levels; a small decrease in high-sensitivity c-reactive protein (hs-CRP) levels; and no difference in fasting blood glucose (FBG), hemoglobin A1c (HbA1c), blood urea nitrogen (BUN), high-density lipoprotein cholesterol (HDL-c), and triglyceride levels in males. Consumption was associated with a small increase in BUN, total cholesterol, HDL-c, and LDL-c levels, a small decrease in hemoglobin, hematocrit, creatinine levels, and no difference in FBG, HbA1c, hs-CRP, and triglyceride levels in females (Table 5 and Table S4). 

### 3.6. Handgrip Strength, Anthropometric Measures, and Blood Pressure

Pork consumption was found to be associated with small increases in handgrip strength, height, weight, body mass index (BMI), systolic blood pressure (SBP), and diastolic blood pressure (DBP), and no difference in waist circumference in males. Consumption was associated with small increase in handgrip strength, height, and weight, a small decrease in BMI, SBP, and DBP, and no difference in waist circumference in females (Table 6 and Table S5). 

## 4. Discussion

To our knowledge, this study is the first to investigate the relationship between pork consumption and total energy and nutrient intake, diet quality, and markers of health status among older Korean adults. We also developed the first nutrient composition database that can be used to estimate the intakes of vitamin B_6_, vitamin B_12_, magnesium, zinc, and selenium from food items reported in the nutrition survey portion of the KNHANES. 

In terms of the contribution of pork to total energy and nutrient intake, our findings in Korea are similar to earlier findings on fresh and lean pork intake in the United States [43,44]. Male and female pork consumers were more likely to meet national recommendations for protein intake compared with non-consumers. For nutrient intake estimation, this study used the nutrient content of foods in their raw state from the food composition database for some foods; this may result in differences in the estimated nutrient intake compared with the nutrient content of the foods in their cooked state [45]. Although pork is a key dietary source of vitamin B_6_ in Korean men [46], vitamin B_6_ intake was shown to be considerably lower in male pork consumers compared with male non-consumers. This could be partly explained by the form in which pork is consumed, along with other specific co-consumed foods within the Korean diet. Lower vitamin B_6_ bioavailability [47] and blood levels have been reported in older adults [48], which may underscore the need to enhance policies and programs to better emphasize foods rich in this nutrient. We observed substantially lower levels of calcium, vitamin A, vitamin C, and vitamin B_6_ relative to the recommendations of the South Korean government. A 2022 study showed underweight Korean older adults to have a lower intake of vitamin A, vitamin C, niacin, and calcium, in which only about 40–70% met the recommended intake [49]. In our study, levels of these nutrients were higher among consumers of pork, albeit lower than the recommended intake. 

The KHEI, similar to the Healthy Eating Index (HEI) used in the United States, was recently designed to assess the diet quality of Korean people [50]. In our study, pork consumers had higher KHEI scores compared with non-consumers. Our data might suggest that pork intake may both (1) directly affect diet quality through increasing the meat, fish, egg, and bean intake component score within the KHEI and (2) indirectly impact diet quality by acting as a carrier food [51] by promoting higher consumption of other healthful components of the Korean diet (e.g., vegetables). Pork is customarily consumed in Korea alongside vegetables and other dishes. 

Our findings among Korean older adults showed small and clinically irrelevant differences in biomarkers of health between pork consumers and non-consumers. We also noted several discrepancies between genders, which were likely influenced by confounders not accounted for in these analyses (or in general observational research) [52,53] and likely reflect type 1 error. Because South Korea has a rapidly aging population, age-related muscular weakness and sarcopenia are becoming major public health concerns. Many factors, including age, ethnicity, gender, and exercise, can affect handgrip strength, which is a recognized general predictor of overall muscle strength. Because of their importance, the Korean Ministry of Health and Welfare has been collecting and incorporating these metrics into the KNHANES since 2014 [54]. As with KHEI scores, male and female pork consumers in this study had significantly stronger handgrip strength than non-consumers. Reports have documented the beneficial effect of animal proteins over plant proteins with regard to improving muscular health. The lower skeletal muscle anabolic properties of plant-based proteins could be attributed to lower digestibility and a lack of specific essential amino acids [55]. Despite the sustainability of plant-source proteins, they are known to have reduced protein quality compared with animal-source protein. Our results are consistent with findings from the United States that suggest fresh and fresh–lean pork is associated with a lower risk of functional impairment among United States adults aged ≥65 years [27]. Increased dietary protein intake have also been associated with handgrip strength in both US and Korean adults [39,56,57,58].

Our study has several strengths and limitations. Its strengths include the use of a large nationally representative cross-sectional dataset. Its limitations include the observational design, which precludes our ability to infer causality. As with all types of observational research, reverse causation and residual confounding may persist. For example, a greater intake of pork may reflect socioeconomic status, which is known to independently influence health. The use of a single 24 h self-reported dietary recall is likely influenced by measurement error and recall bias, the latter having been shown to be even more predominant in older populations [59]. A 24 h dietary recall may also not reflect the overall dietary pattern of an individual. Small, clinically irrelevant, differences in measured biomarkers of health are reflective of the high sample size and might be indicate an increased likelihood of type-1 error. Finally, because this study was conducted using only data for Korean older adults, the conclusions are not generalizable to other populations and cultures. 

## 5. Conclusions

Older adults are generally more likely to be nutritionally vulnerable compared with other life stages. The results of this study suggest that pork consumption may subtly contribute to higher intake of energy and most nutrients, improvement of diet quality scores, and higher handgrip strength among Korean older adults. However, the study conclusions cannot be generalizable to other populations due to large differences in consumption patterns between cultures. Future well-designed clinical studies are needed to confirm our findings 

## Figures and Tables

**Table 1:** General characteristics of participants according to pork consumption by gender, 2016–2020 KNHANES.

Characteristic	All (*n* = 2068)	Men		Women	
		Consumers (*n* = 372)	Non-Consumers (*n* = 618)	Consumers (*n* = 359)	Non-Consumers (*n* = 719)
Age (y)	72.28 ± 0.13	71.65 ± 0.32	73.35 ± 0.23 ****	71.15 ± 0.32	72.26 ± 0.23 **
Education					
Elementary school	1013 (50.39)	119 (32.20)	251 (42.14) **	194 (52.05)	449 (66.88) **
Middle school	253 (14.29)	38 (11.41)	82 (15.8)	56 (18.54)	77 (12.27)
High school	353 (20.33)	89 (27.81)	120 (22.02)	51 (18.93)	93 (15.45)
College	251 (14.99)	92 (28.57)	97 (20.04)	29 (10.48)	33 (5.40)
Residence					
Urban	1484 (77.09)	289 (83.30)	433 (74.93) *	275 (81.35)	487 (73.25) *
Rural	584 (22.91)	83 (16.70)	185 (25.07)	84 (18.65)	232 (26.75)
Household income					
Low	910 (42.71)	117 (31.89)	268 (42.67) **	151 (41.32)	374 (49.64)
Middle-low	607 (28.70)	119 (30.79)	188 (29.68)	115 (30.73)	185 (25.51)
Middle-high	311 (16.42)	74 (20.04)	101 (17.62)	46 (14.63)	90 (14.24)
High	228 (12.17)	60 (17.29)	58 (10.04)	45 (13.33)	65 (10.61)
Marital status					
Married	2060 (99.62)	370 (99.48)	617 (99.9)	357 (99.45)	716 (99.53)
Single	8 (0.38)	2 (0.52)	1 (0.10)	2 (0.55)	3 (0.47)
Alcohol consumer					
No	1278 (59.96)	142 (36.31)	296 (47.66) **	279 (75.52)	561 (76.35)
Yes	748 (37.96)	226 (62.44)	312 (50.73)	72 (21.95)	138 (20.91)
No response	42 (2.08)	4 (1.25)	10 (1.61)	8 (2.53)	20 (2.75)
Smoking status					
Current smoker	214 (10.78)	84 (22.32)	100 (15.71)	9 (3.30)	21 (3.71)
Ex-smoker	599 (29.81)	203 (55.14)	360 (58.00)	11 (3.80)	25 (3.42)
Non-smoker	1212 (57.26)	81 (21.30)	147 (24.48)	331 (90.37)	653 (90.08)
No response	43 (2.16)	4 (1.25)	11 (1.81)	8 (2.53)	20 (2.79)
Regular physical activity					
No	1223 (64.29)	206 (57.98)	347 (61.86)	217 (64.27)	453 (69.94)
Yes	637 (35.71)	129 (42.02)	200 (38.14)	113 (35.73)	195 (30.06)

KNHANES = Korean National Health and Nutrition Examination Survey. Values are presented as the mean ± SE. The Rao–Scott chi-square test was used considering the complex survey design to compare the characteristics across the categorical variables. * *p* < 0.05, ** *p* < 0.01, and **** *p* < 0.0001 between pork consumers and non-consumers.

**Table 2:** Intake of fresh pork, processed pork, and total pork.

Characteristic	*n*	Intake, Mean ± SE (g/d)		
		Total Pork	Fresh Pork	Processed Pork
Total population	731	63.11 ± 3.16	60.46 ± 3.19	2.65 ± 0.36
Gender				
Men	372	79.11 ± 5.17	75.99 ± 5.24	3.12 ± 0.64
Women	359	46.30 ± 2.87	44.15 ± 2.91	2.15 ± 0.32
BMI (kg/m^2^)				
Underweight (<18.5)	37	50.42 ± 9.84	48.03 ± 9.91	2.39 ± 1.61
Healthy weight (18.5 to <25.0)	351	59.41 ± 4.24	56.92 ± 4.29	2.50 ± 0.50
Overweight (25 to <30.0)	195	63.85 ± 5.70	60.88 ± 5.80	2.97 ± 0.67
Obese (≥30.0)	138	74.64 ± 9.06	71.88 ± 9.14	2.75 ± 1.02

BMI = body mass index.

**Table 3:** Nutrient intake between pork consumers and non-consumers by gender in the healthy Korean population aged 65 years or older, per the 2016–2020 KNHANES.

Nutrient	All (*n* = 2068)	Men			Women		
		Consumers (*n* = 372)	Non-Consumers (*n* = 618)	*p*-Value	Consumers (*n* = 359)	Non-Consumers (*n* = 719)	*p*-Value
Energy (kcal)	1706.45 ± 17.16	2080.79 ± 43.18	1816.08 ± 30.37	<0.0001	1601.93 ± 35.11	1449.33 ± 23.48	<0.0001
Carbohydrate (g)	290.59 ± 2.92	327.06 ± 7.30	312.37 ± 5.31	0.0011	267.70 ± 6.02	262.14 ± 4.56	0.0053
Protein (g)	57.75 ± 0.73	74.49 ± 1.80	61.37 ± 1.33	<0.0001	56.15 ± 1.29	45.77 ± 0.90	<0.0001
Fat (g)	29.73 ± 0.62	40.27 ± 1.53	28.88 ± 0.96	<0.0001	32.63 ± 1.43	22.98 ± 0.77	<0.0001
SFA (g)	9.11 ± 0.21	12.43 ± 0.46	8.68 ± 0.32	<0.0001	10.15 ± 0.55	7.06 ± 0.26	<0.0001
ω3 fatty acids (g)	1.67 ± 0.06	1.95 ± 0.14	1.80 ± 0.09	0.1025	1.86 ± 0.17	1.29 ± 0.06	<0.0001
ω6 fatty acids (g)	6.89 ± 0.15	9.12 ± 0.40	6.69 ± 0.22	<0.0001	7.53 ± 0.35	5.46 ± 0.21	<0.0001
Cholesterol (mg)	160.54 ± 4.43	212.45 ± 10.65	166.30 ± 7.87	<0.0001	160.70 ± 8.32	125.70 ± 6.52	<0.0001
Sugar (g)	53.95 ± 1.12	59.53 ± 2.40	54.54 ± 1.98	<0.0001	56.51 ± 2.59	48.85 ± 1.78	<0.0001
Calcium (mg)	469.92 ± 8.11	517.55 ± 14.84	515.27 ± 15.03	0.0030	454.96 ± 15.80	409.07 ± 11.82	<0.0001
Phosphorus (mg)	946.25 ± 11.21	1142.27 ± 24.86	1019.79 ± 20.56	<0.0001	913.27 ± 21.04	784.94 ± 14.76	<0.0001
Iron (mg)	10.46 ± 0.17	12.36 ± 0.49	11.30 ± 0.33	0.0005	9.77 ± 0.32	8.97 ± 0.22	0.0008
Sodium (mg)	2936.37 ± 47.90	3611.58 ± 114.99	3234.82 ± 92.63	0.0028	2685.48 ± 77.24	2413.14 ± 74.46	<0.0001
Potassium (mg)	2709.23 ± 38.61	3136.77 ± 79.96	2859.60 ± 68.05	<0.0001	2673.37 ± 77.82	2347.22 ± 57.35	<0.0001
Vitamin A (µg RAE)	335.50 ± 11.16	406.76 ± 42.80	322.47 ± 13.26	0.0252	365.13 ± 22.44	291.14 ± 12.93	0.0001
Carotene (μg)	2898.12 ± 85.02	3215.74 ± 190.08	2920.45 ± 147.59	0.0012	3156.32 ± 204.43	2558.07 ± 128.67	0.0456
Retinol (μg)	93.95 ± 8.28	138.81 ± 39.67	79.09 ± 4.80	0.1213	102.11 ± 10.69	77.85 ± 6.88	0.0244
Thiamin(mg)	1.11 ± 0.01	1.46 ± 0.04	1.09 ± 0.02	<0.0001	1.15 ± 0.03	0.92 ± 0.02	<0.0001
Riboflavin(mg)	1.24 ± 0.02	1.51 ± 0.05	1.29 ± 0.03	<0.0001	1.25 ± 0.04	1.03 ± 0.03	<0.0001
Niacin (mg)	10.53 ± 0.14	13.39 ± 0.34	11.13 ± 0.28	<0.0001	10.15 ± 0.26	8.55 ± 0.19	<0.0001
Folate (μg DFE)	326.25 ± 4.87	369.18 ± 9.48	355.38 ± 9.37	0.0008	312.43 ± 9.54	282.44 ± 6.51	<0.0001
Vitamin C (mg)	63.51 ± 1.96	69.02 ± 4.63	61.66 ± 2.71	0.0007	70.33 ± 4.94	58.43 ± 3.36	0.0004
Vitamin B_6_ (mg)	0.92 ± 0.04	1.13 ± 0.09	1.03 ± 0.07	0.5481	0.83 ± 0.06	0.76 ± 0.06	0.0689
Vitamin B_12_ (μg)	4.58 ± 0.18	4.88 ± 0.31	5.56 ± 0.37	0.0085	4.29 ± 0.45	3.67 ± 0.22	0.0010
Magnesium (mg)	301.26 ± 3.78	345.58 ± 8.69	329.88 ± 7.11	<0.0001	285.81 ± 7.51	258.04 ± 5.16	<0.0001
Zinc (mg)	11.59 ± 0.21	13.10 ± 0.37	12.54 ± 0.42	0.1170	11.06 ± 0.38	10.15 ± 0.30	0.0006
Selenium (μg)	67.52 ± 1.48	89.59 ± 3.40	66.65 ± 2.09	<0.0001	70.26 ± 2.82	54.35 ± 2.42	<0.0001
Fiber (g)	27.31 ± 0.45	30.11 ± 0.86	29.25 ± 0.79	0.0084	26.69 ± 0.83	24.26 ± 0.65	<0.0001

DFE = dietary folate equivalents; KNHANES = Korean National Health and Nutrition Examination Survey; RAE = retinol activity equivalents. Values are presented as the mean ± SE. *p*-values have been adjusted by age.

**Table 4:** KHEI score and component scores between pork consumers and non-consumers by gender in the healthy Korean population aged ≥65 years, per the 2016–2018 KNHANES.

KHEI Score and Components	All (*n* = 1254)	Men			Women		
		Consumers (*n* = 235)	Non-Consumers (*n* = 363)	*p*- Value	Consumers (*n* = 207)	Non-Consumers (*n* = 449)	*p*- Value
KHEI score	67.46 ± 0.46	67.91 ± 0.93	65.74 ± 0.74	0.0308	70.88 ± 0.96	67.00 ± 0.73	<0.0001
KHEI component							
Meat, fish, egg, and bean intake (serving/day)	6.78 ± 0.12	7.58 ± 0.22	6.49 ± 0.20	<0.0001	7.93 ± 0.22	6.03 ± 0.19	<0.0001
Have breakfast (times/week)	9.43 ± 0.08	9.51 ± 0.15	9.59 ± 0.11	0.0117	9.53 ± 0.19	9.20 ± 0.14	0.0281
Mixed grains intake (serving/day)	2.50 ± 0.08	2.42 ± 0.17	2.61 ± 0.13	0.2554	2.50 ± 0.17	2.45 ± 0.13	0.4235
Total fruit intake (serving/day)	2.80 ± 0.09	2.57 ± 0.18	2.43 ± 0.15	0.1579	3.21 ± 0.18	3.04 ± 0.16	<0.0001
Fresh fruit intake (serving/day)	2.89 ± 0.09	2.68 ± 0.19	2.62 ± 0.16	0.6017	3.29 ± 0.18	3.05 ± 0.16	0.001
Total vegetable intake (serving/day)	3.74 ± 0.05	4.07 ± 0.09	3.61 ± 0.09	0.0003	3.86 ± 0.10	3.59 ± 0.09	<0.0001
Vegetable intake excluding Kimchi and pickled vegetable intake (serving/day)	3.32 ± 0.06	3.31 ± 0.12	3.02 ± 0.09	0.011	3.72 ± 0.13	3.37 ± 0.09	<0.0001
Milk and milk products intake (serving/day)	2.62 ± 0.15	2.49 ± 0.34	2.15 ± 0.23	0.2861	2.76 ± 0.33	3.02 ± 0.26	0.0220
Percent energy from SFA (% of total energy intake)	9.04 ± 0.08	8.88 ± 0.18	9.26 ± 0.12	0.2728	8.36 ± 0.27	9.26 ± 0.11	0.0155
Sodium intake (mg/day)	7.66 ± 0.10	6.44 ± 0.25	7.25 ± 0.17	0.0051	7.96 ± 0.20	8.52 ± 0.14	<0.0001
Percent energy from sweets and beverages (% of total energy intake)	9.36 ± 0.07	9.33 ± 0.15	9.23 ± 0.15	0.4244	9.52 ± 0.13	9.40 ± 0.12	0.6741
Percent energy from carbohydrate (% of total energy intake)	1.63 ± 0.07	2.21 ± 0.16	1.60 ± 0.11	0.0028	2.16 ± 0.18	1.09 ± 0.09	<0.0001
Percent energy intake from fat (% of total energy intake)	2.46 ± 0.08	3.20 ± 0.16	2.39 ± 0.13	0.0004	2.99 ± 0.20	1.86 ± 0.12	<0.0001
Energy intake (% of the EER)	3.24 ± 0.07	3.22 ± 0.18	3.49 ± 0.12	0.4786	3.09 ± 0.18	3.12 ± 0.11	0.6345

EER = energy requirement; KHEI = Korean Healthy Eating Index; KNHANES = Korean National Health and Nutrition Examination Survey; SFA = saturated fatty acid. Note: KHEI data were released for 2016–2018 KNHANES. Values are presented as the mean ± SE. *p*-values have been adjusted by age.

**Table 5:** Blood biomarkers of health status between pork consumers and non-consumers by gender in the healthy Korean population aged 65 years old and over, 2016–2020 KNHANES.

Blood Biomarkers	All	Men			Women		
		Consumers	Non-Consumers	*p*-Value	Consumers	Non-Consumers	*p*-Value
Hemoglobin (g/dL)	*n* = 1966	*n* = 358	*n* = 588		*n* = 343	*n* = 677	
	13.81 ± 0.04	14.69 ± 0.08	14.50 ± 0.07	<0.0001	13.04 ± 0.07	13.09 ± 0.04	0.0003
Hematocrit (%)	*n* = 1966	*n* = 358	*n* = 588		*n* = 343	*n* = 677	
	42.07 ± 0.12	44.44 ± 0.24	43.92 ± 0.22	<0.0001	39.95 ± 0.21	40.14 ± 0.13	0.0031
FBG (mg/dL)	*n* = 1971	*n* = 359	*n* = 588		*n* = 346	*n* = 678	
	100.05 ± 0.44	100.74 ± 1.02	102.15 ± 0.87	0.3373	99.13 ± 0.99	98.20 ± 0.68	0.0957
HbA_1c_ (%)	*n* = 1966	*n* = 358	*n* = 588		*n* = 343	*n* = 677	
	5.74 ± 0.01	5.72 ± 0.04	5.76 ± 0.03	0.6393	5.76 ± 0.04	5.72 ± 0.02	0.4849
hs-CRP (mg/L)	*n* = 1159	*n* = 221	*n* = 336		*n* = 199	*n* = 403	
	1.45 ± 0.10	1.55 ± 0.23	1.73 ± 0.20 **	0.0252	1.04 ± 0.08	1.37 ± 0.18	0.135
Creatinine (mg/dL)	*n* = 1971	*n* = 359	*n* = 588		*n* = 346	*n* = 678	
	0.82 ± 0.01	0.94 ± 0.01	0.93 ± 0.01	0.0011	0.69 ± 0.01	0.71 ± 0.01	<.0001
BUN (mg/dL)	*n* = 1971	*n* = 359	*n* = 588		*n* = 346	*n* = 678	
	16.69 ± 0.14	17.17 ± 0.27	17.13 ± 0.32	0.1566	16.37 ± 0.26	16.18 ± 0.21	0.0379
TC (mg/dL)	*n* = 1971	*n* = 359	*n* = 588		*n* = 346	*n* = 678	
	200.23 ± 0.94	193.43 ± 1.96	190.50 ± 1.67	0.0057	211.87 ± 2.14	206.79 ± 1.65	0.0011
HDL-c (mg/dL)	*n* = 1971	*n* = 359	*n* = 588		*n* = 346	*n* = 678	
	49.78 ± 0.33	48.38 ± 0.73	47.77 ± 0.57	0.2112	53.10 ± 0.77	50.60 ± 0.56	0.0399
LDL-c (mg/dL)	*n* = 1971	*n* = 359	*n* = 588		*n* = 346	*n* = 678	
	125.54 ± 0.82	119.29 ± 1.67	118.33 ± 1.41	0.0371	134.12 ± 1.98	131.12 ±1.47	0.0006
TG (mg/dL)	*n* = 1971	*n* = 359	*n* = 588		*n* = 346	*n* = 678	
	124.59 ± 1.88	128.79 ± 4.40	122.02 ± 3.15	0.1005	123.20 ± 3.78	125.30 ± 3.49	0.2649

BUN = blood urea nitrogen; HbA1c = glycated hemoglobin; hs-CRP = high-sensitivity C-reactive protein; HDL-c = high-density lipoprotein cholesterol; KNHANES = Korean National Health and Nutrition Examination Survey; LDL-c = low-density lipoprotein cholesterol; TC = total cholesterol; TG = triglycerides. hs-CRP was not measured in 2019 and 2020. Note: Values are presented as the mean ± SE. *p*-values have been adjusted by age. Significant difference at ** *p* <0.01 between pork consumers and non-consumers.

**Table 6:** Hand grip strength, anthropometric measurements, and blood pressure between pork consumers and non-consumers by gender in the healthy Korean population aged 65 years or older, 2016–2020 KNHANES.

Measurement	All	Men			Women		
		Consumers	Non-Consumers	*p*-Value	Consumers	Non-Consumers	*p*-Value
Hand grip strength (kg)	*n* = 1600	*n* = 290	*n* = 477		*n* = 271	*n* = 562	
	26.32 ± 0.26	33.84 ± 0.52	31.91 ± 0.40	<0.0001	20.76 ± 0.34	19.99 ± 0.22	<0.0001
BMI (kg/m^2^)	*n* = 2041	*n* = 367	*n* = 612		*n* = 354	*n* = 708	
	23.01 ± 0.08	22.95 ± 0.18	22.57 ± 0.14	0.0004	23.24 ± 0.18	23.34 ± 0.15	0.0108
Waist circumference (cm)	*n* = 2062	*n* = 371	*n* = 617		*n* = 358	*n* = 716	
	83.06 ± 0.24	85.31 ± 0.53	84.06 ± 0.42	0.2621	81.47 ± 0.51	81.71 ± 0.43	0.8723
Blood pressure	*n* = 2059	*n* = 370	*n* = 618		*n* = 358	*n* = 713	
SBP (mm Hg)	126.84 ± 0.50	126.27 ± 0.98	126.05 ± 0.83	<0.0001	122.28 ± 0.22	128.18 ± 0.87	<0.0001
DBP (mm Hg)	73.62 ± 0.27	73.50 ± 0.66	73.15 ± 0.45	<0.0001	72.48 ± 0.14	73.45 ± 0.46	0.0029

BMI = body mass index; DBP = diastolic blood pressure; KNHANES = Korean National Health and Nutrition Examination Survey; SBP = systolic blood pressure. Note: Hand grip strength was not measured due to COVID-19 in 2020. Values are presented as the mean ± SE. *p*-values have been adjusted by age.

## Data Availability

Datasets are available on request: The raw data supporting the conclusions of this article will be made available by the authors, without undue reservation.

## Supplementary Materials

The following supporting information can be downloaded at https://www.mdpi.com/article/10.3390/nu16234188/s1, Table S1: Developing the value of vitamins B_6_ and B_12_, magnesium, zinc, and selenium using representative Korean, Japan, and U.S. databases; Table S2: Nutrient intake between pork consumers and pork non-consumers in the healthy elderly aged 65 years old and over, KNHANES 2016–2020; Table S3: KHEI score and components between pork consumers and pork non-consumers in the healthy elderly aged 65 years old and over, KNHANES 2016–2018; Table S4: Blood biomarkers of health status between pork consumers and non-consumers in the healthy elderly aged 65 years old and over, KNHANES 2016–2020; Table S5: Hand grip strength, anthropometric measurements and blood pressure between pork consumers and pork non-consumers in the healthy elderly aged 65 years old and over, KNHANES 2016–2020.
