# Supplementary material for: The Relationship of Pork Meat Consumption with Nutrient Intakes, Diet Quality, and Biomarkers of Health Status in Korean Older Adults
Source: Nutrients. 2024 Dec 4;16(23):4188. doi: 10.3390/nu16234188 (PMC11644605; doi:10.3390/nu16234188)
Supplement: Supplementary file 1 [file nutrients-16-04188-s001.zip › nutrients-3350590-supplementary.pdf]

Supplementary Material

Table S1: Developing reference values for vitamins B_6 and B_{12}, magnesium, zinc, and selenium using representative Korean, Japanese, and U.S. food composition databases.

	Food composition table, RDA	Food Nutrients Database Processed Food, KFDA	Japanese Standard tables of food composition, MEXT	Food Data Central, USDA SR28	CAN-Pro, The Korean Nutrition Society
**Vitamin B_6**					
Exact same[1]	329	-	-	-	-
Similar food[2]	459	1	51	8	464
Imputation[3]	35	-	-	-	-
Moisture adjusted	12	-	-	-	-
Total	835	1	51	8	464
**Vitamin B_{12}**					
Exact same[1]	986	-	-	-	-
Similar food[2]	2,026	-	47	7	257
Imputation[3]	111	-	-	-	-
Moisture adjusted	29	-	-	-	-
Total	3,134	-	47	7	257
**Magnesium**					
Exact same[1]	1,008	-	-	-	-
Similar food[2]	2,078	16	100	8	257
Imputation[3]	115	-	-	-	-
Moisture adjusted	29	-	-	-	-
Total	3,230	16	100	8	257
**Zinc**					
Exact same[1]	997	-	-	-	-
Similar food[2]	2,071	3	102	8	257
Imputation[3]	114	-	-	-	-

Moisture adjusted	29	-	-	-	-
Total	3,211	3	102	8	257
**Selenium**					
Exact same[1]	961	-	-	-	-
Similar food[2]	1,992	1	63	8	257
Imputation[3]	111	-	-	-	-
Moisture adjusted	29	-	-	-	-
Total	3,093	1	63	8	257

[1] Foods with same description (exact matching names and same condition)

[2] Foods with the same name but different water contents (dried, raw, and boiled) or different manufacturer

[3] Substitute similar foods (carbohydrate, protein, and fat contents were taken into account)

Table S2: Nutrient intakes among consumers and nonconsumers of pork in the healthy elderly Korean population, KNHANES 2016 - 2020.

Nutrients	Consumers (*n* =731)			Non-consumers (*n* =1,337)		
Energy (kcal)	1847.22	±	29.7****	1625.50	±	21.07
Carbohydrate (g)	298.11	±	4.83****	286.27	±	3.76
Protein (g)	65.55	±	1.17****	53.26	±	0.87
Fat (g)	36.55	±	1.14****	25.82	±	0.66
SFA (g)	11.32	±	0.37****	7.84	±	0.22
ω3 (g)	1.91	±	0.13****	1.53	±	0.06
ω6 (g)	8.34	±	0.28****	6.05	±	0.16
Cholesterol (mg)	187.21	±	6.76****	145.20	±	5.48
Sugar (g)	58.06	±	1.82****	51.59	±	1.41
Calcium (mg)	487.02	±	11.41****	460.08	±	10.61
Phosphorus (mg)	1030.58	±	17.28****	897.75	±	13.87
Iron (mg)	11.10	±	0.30****	10.09	±	0.21
Sodium (mg)	3159.87	±	72.27****	2807.84	±	62.54
Potassium (mg)	2910.75	±	58.48****	2593.35	±	46.83
Vitamin A (μg RE)	611.83	±	35.45***	518.76	±	21.23
Carotene (μg)	3186.76	±	146.81***	2732.15	±	100.44
Retinol (μg)	120.91	±	21.08*	78.45	±	4.35
Thiamin (mg)	1.31	±	0.02****	1.00	±	0.02
Riboflavin (mg)	1.38	±	0.03****	1.16	±	0.02
Niacin (mg)	11.81	±	0.24****	9.79	±	0.17
Folate (μg DFE)	341.50	±	7.01****	317.48	±	6.17
Vitamin C (mg)	69.66	±	3.46****	59.98	±	2.28
Vitamin B6 (mg)	0.98	±	0.06***	0.89	±	0.05
Vitamin B12 (μg)	4.59	±	0.27****	4.58	±	0.23
Magnesium (mg)	316.43	±	6.15****	292.54	±	4.66
Zinc (mg)	12.10	±	0.28****	11.30	±	0.28
Selenium (μg)	80.16	±	2.33****	60.26	±	1.74

Table S3: KHEI scores and component scores among healthy elderly consumers and nonconsumers of pork, KNHANES 2016–2018.

KHEI score and components	Consumers (n = 442)	Non-consumers (n = 812)
KHEI score	69.29 ± 0.68****	66.43 ± 0.55
KHEI components		
Meat, fish, eggs and beans intake (serving/day)	7.74 ± 0.16****	6.24 ± 0.15
Have breakfast (times/week)	9.52 ± 0.12	9.38 ± 0.09
Mixed grains intake (serving/day)	2.45 ± 0.13	2.52 ± 0.10
Total fruits intake (serving/day)	2.87 ± 0.13****	2.76 ± 0.11
Fresh fruits intake (serving/day)	2.96 ± 0.14****	2.85 ± 0.12
Total vegetables intake (serving/day)	3.97 ± 0.07****	3.60 ± 0.07
Vegetables intake excluding Kimchi and pickled vegetables intake (serving/day)	3.50 ± 0.10****	3.21 ± 0.07
Milk and milk products intake (serving/day)	2.61 ± 0.26	2.63 ± 0.17*
Percentage of energy from saturated fatty acid (% of total energy intake)	8.64 ± 0.16	9.26 ± 0.08**
Sodium intake (mg/day)	7.15 ± 0.16	7.95 ± 0.11****
Percentage of energy from sweets and beverages (% of total energy intake)	9.42 ± 0.10	9.32 ± 0.10
Percentage of energy from carbohydrate (% of total energy intake)	2.19 ± 0.13****	1.32 ± 0.07
Percentage of energy intake from fat (% of total energy intake)	3.10 ± 0.13****	2.10 ± 0.09
Energy intake (% of the EER)	3.16 ± 0.12	3.29 ± 0.09

Note: 2019 and 2020 KHEI data to be released in 2023.

Values were presented as the mean ± standard error. *P*-values were adjusted for age and gender. Significantly different at *$p<0.05$, **$p<0.01$, and ****$p<0.0001$ between pork consumers and pork non-consumers. KHEI: Korean healthy eating index; KNHANES: Korean National Health and Nutrition Examination Survey.

Table S4: Blood biomarker levels of elderly consumers and nonconsumers of pork, KNHANES 2016–2020.

Blood biomarkers	Consumers	Non-consumers
Hemoglobin (g/dL)	*n* = 701	*n* =1,265
	13.89 ± 0.07****	13.77 ± 0.05
Hematocrit (%)	*n* = 701	*n* =1,265
	42.26 ± 0.19****	41.96 ± 0.14
Fasting blood glucose (mg/dL)	*n* = 705	*n* =1,266
	99.96 ± 0.71	100.10 ± 0.57**
HbA1c (%)	*n* = 701	*n* =1,265
	5.74 ± 0.03	5.74 ± 0.02
hs-CRP (mg/L)	*n* = 420	*n* = 739
	1.31 ± 0.13	1.54 ± 0.13**
Creatinine (mg/dL)	*n* = 705	*n* = 1,266
	0.82 ± 0.01	0.82 ± 0.01
BUN (mg/dL)	*n* = 705	*n* =1,266
	16.78 ± 0.19****	16.64 ± 0.20
Total cholesterol (mg/dL)	*n* = 705	*n* = 1,266
	202.41 ± 1.45****	198.95 ± 1.22
HDL-cholesterol (mg/dL)	*n* = 705	*n* = 1,266
	50.68 ± 0.55****	49.24 ± 0.40
LDL-cholesterol (mg/dL)	*n* = 705	*n* = 1,266
	126.51 ± 1.32****	124.96 ± 1.04
Triglycerides (mg/dL)	*n* = 705	*n* = 1,266
	126.07 ± 2.90	123.72 ± 2.38

Note: hs-CRP was not measured in 2019 and 2020.

Values were presented as the mean ± standard error. *p*-values were adjusted for age and gender. Significantly different at $^{**}p<0.01$ and $^{****}p<0.0001$ between pork consumers and pork non-consumers.

HbA1c: glycated hemoglobin; hs-CRP: high-sensitivity C-reactive protein; HDL-cholesterol: high-density lipoprotein cholesterol; LDL-cholesterol: low-density lipoprotein cholesterol; BUN: blood urea nitrogen; KNHANES: Korean National Health and Nutrition Examination Survey.

Table S5: Hand grip strength, anthropometric measures, and blood pressure among healthy elderly consumers and nonconsumers of pork, KNHANES 2016–2020.

	**Consumers**	**Non-consumers**
Hand grip strength (kg)	*n* = 561	*n* =1,039
	27.57 ± 0.42****	25.62 ± 0.32
Height (cm)	*n* = 721	*n* = 1,320
	160.40 ± 0.39****	158.45 ± 0.30
Weight (kg)	*n* = 730	*n* =1,334
	59.54 ± 0.45****	57.73 ± 0.32
BMI (kg/m^2)	*n* = 721	*n* =1,320
	23.09 ± 0.13****	22.97 ± 0.10
Waist circumference (cm)	*n* =729	*n* = 1,333
	83.44 ± 0.40****	82.84 ± 0.30
Blood pressure (mmHg)	*n* =728	*n* = 1,331
Systolic blood pressure (mmHg)	126.29 ± 0.71	127.15 ± 0.64****
Diastolic blood pressure (mmHg)	74.17 ± 0.44	74.73 ± 0.13****

Values were presented as the mean ± standard error. *p*-values were adjusted for age and gender. Significantly different at ****p<0.0001 between pork consumers and pork non-consumers. KNHANES: Korean National Health and Nutrition Examination Survey; BMI: body mass index.
